# Editorial: Antifungal Drug Discovery: New Theories and New Therapies

**DOI:** 10.3389/fmicb.2016.00728

**Published:** 2016-05-23

**Authors:** Chaminda J. Seneviratne, Edvaldo A. R. Rosa

**Affiliations:** ^1^Faculty of Dentistry, National University of SingaporeSingapore, Singapore; ^2^The Pontifical Catholic University of ParanáCuritiba, Brazil

**Keywords:** antifungals, *Candida albicans*, new drug discovery, oral candidiasis, Candida biofilms

Medically important fungal infections can be broadly classified into superficial surface infections and invasive mycoses (Samaranayake and MacFarlane, 1990; Roemer and Krysan, 2014). Superficial surface infections include mucosal candidiasis, dermatophyte infections whereas invasive mycoses affect sterile body sites such as bloodstream, central nervous system, kidney, lungs, and liver. Rise of fungal infections has caused a substantial morbidity and mortality globally (Vallabhaneni et al., 2016). It is reported that mortality among patients with invasive candidiasis is as high as 40%, even when patients receive antifungal therapy (Kullberg and Arendrup, 2015).

Antifungal drugs are relatively difficult to develop compared to antibacterial drugs owing to the eukaryotic nature of the cells. Only a few classes of antifungal drugs, such as polyenes, azoles, echinocandins, allylamines, and flucytosine, are available to treat the myriad of fungal infections (Sanglard et al., 2009). Of the current antifungal agents, none have all the characteristics of an ideal agent (Wong et al., 2014). Antifungal resistance and host-related adverse reactions further limit the existing antifungal arsenal against fungal pathogens (Chandrasekar, 2011). Rising drug resistance is an inevitable problem, particularly for fluconazole, a drug of choice for candidiasis in AIDS patients (Siikala et al., 2010; Rautemaa and Ramage, 2011). Drug resistance has also been reported for recently introduced echinocandin antifungal agents (Seneviratne et al., 2008a; Ben-Ami et al., 2011; Clancy and Nguyen, 2011). Moreover, some fungal species are inherently resistance to existing antifungals (Sanglard; Kołaczkowska and Kołaczkowski, 2016). In addition, biofilm mode of fungal growth is known to be highly resistant to antifungal agents (Chandra et al., 2005; Seneviratne et al., 2008b). Hence, the development of more effective and safe antifungal agents is a top priority in the health care field. Therefore, this special research topic aimed to address the new theories and therapies pertaining to antifungal drug discovery, covering aspects of clinical relevance and novel antifungal strategies.

Majority of the articles published under this research topic belongs to the *Candida* species, which is a group of major fungal pathogens in humans. *Candida* species are commensal fungi that inhabit various niches of the human body, including the oral cavity, gastrointestinal tract, vagina, and skin (Samaranayake and MacFarlane, 1990; Mayer et al., 2013). However, under certain circumstances, *Candida* can cause infections, or candidiasis, ranging from superficial mucous membrane infections to life-threatening systemic diseases. *Candida albicans* is the most prevalent fungal pathogen in lethal blood stream infections of humans (Seneviratne et al., 2011). *C. albicans* infections are a significant clinical problem especially in compromised host populations undergoing HIV/AIDS treatment, chemotherapy or organ transplantation. Moreover, sharp increase in aging populations which are susceptible to fungal infections is expected in the next few decades. The currently available antifungal agents are not always effective against *C. albicans*, which remains a ubiquitous pathogen in nosocomial diseases, causing severe mucosal infections such as oral candidiasis, onycomycoses, vulvovaginal candidiasis, and systemic mycoses with high mortality rates (Kojic and Darouiche, 2004; Zaoutis et al., 2005; Concia et al., 2009).

At the start of the research topic, clinical relevance of oral candidiasis has been discussed in order to provide a glimpse of human fungal infections (Patil et al.). Biofilm formation of the fungal pathogen is a significant problem in medical-device associated infections and directly related to therapeutic failure (Williams and Ramage, 2015). As conventional antifungal agents are ineffective against fungal biofilms, alternative strategies are needed. Novel antifungal compounds that target fungal biofilm formation and the host inflammatory response such as myriocin, fulvic acid, and acetylcholine have been discussed in the research topic as candidate dual action therapeutics to treat opportunistic fungal infections (Borghi et al.). Microbial biotransformation has emerged as an important tool for obtaining novel substances which possess antifungal activity. Implication of endophytic fungi as cell factories for producing new antifungal molecules and *in silico* approach using databases of 3D molecular structures are also discussed (Bianchini et al.). Oshima and colleagues introduce an interesting concept of biogenics for oral candidiasis. Biogenics advocates the use of beneficial bioactive substances produced by probiotic bacteria, whose activities are independent of the viability of probiotic bacteria in human bodies. Ravikumar and colleagues examine various immune-enhancing strategies for the invasive fungal diseases caused by *Candida* and *Aspergillus* species. These novel approaches include cytokine therapy, granulocyte transfusion, antibody-based therapy, natural killer cell treatment and adoptive T cell transfer. Molecules such as phenolic compounds, derived from natural sources and exhibiting considerable antifungal properties are a source for the development of novel anti-candidal therapy (Teodoro et al.). Therefore, potential use, proposed mechanisms of action and limitations of phenolic acids have been discussed.

*Candida* bloodstream isolates derived from Hong Kong have shown to possess virulence attributes such as biofilm formation, hemolysin production, proteinase activity as well as perturbations in their antifungal sensitivity in the presence of serum, which may contribute to treatment complication in candidemia (Seneviratne et al.). One of the major mechanisms contributing to multi-drug resistance in *C. albicans* is the plasma membrane drug-efflux system. Therefore, application of inhibitors of drug-efflux pumps has been suggested as a strategy to increase the susceptibility of *C. albicans* to antifungals. Szczepaniak et al. developed a new fluorescence method that allows *in vivo* activity evaluation of compounds inhibiting *C. albicans* transporters. They demonstrated that fluorescence labeling with diS-C3(3) potentiometric dye enables a real-time observation of the activity of *C. albicans* Cdr1 and Cdr2 transporters. The new method was able to demonstrate the different specificities of enniatin A and beauvericin toward drug-efflux pumps. In another study investigators have developed three structurally related chemo-sensititzers i.e., oxathiolone fused chalcone derivatives to successfully restore the sensitivity of fluconazole resistant *C. albicans* strains. The mechanism of action is a possible non-competitive inhibition of drug-efflux pumps Mdr1, Cdr1, and Cdr2. However, more research is warranted in this area to fully establish the role of chemo-sensitizers in clinical use.

Antimicrobial peptide isolates from various sources are also a promising source to develop novel antimycotic agents. A study under this research topic has shown anti-*Candida* activity of antimicrobial peptide produced by *Enterococcus faecium* (Roy et al.). It appears to target chitin in the cell wall of *Candida* species. Host derived molecules like histatin 5 protects human oral mucosa against the transformation of commensal *C. albicans* into a pathogenic invader. A work by Moffa and colleagues demonstrated that coating with histatin 5 reduces *C. albicans* colonization of epithelial cell surfaces and also protects the basal cell layers from undergoing apoptosis. Hence, there is a possibility of using host derived antifungal molecules to prevent *Candida* infections, which may be a useful strategy in compromised host populations.

*Candida glabrata* is an emerging human fungal pathogen. A study examined the role of glucose sensing mechanism in *C. glabrata* using SNF3 (Sucrose Non Fermenting 3) knockout strains. Mutation results in higher susceptibility to amphotericin B in low glucose environment (0.1%), but showed no effect on biofilm formation capability. Going beyond *Candida* species, a study of dermatophyte fungus *Trichophyton rubrum* investigated the role of Hsp90 in its pathogenicity and drug susceptibility. Chemical inhibition of Hsp90 resulted in increased susceptibility of the fungus to itraconazole and micafungin. The synergism observed between the inhibition of Hsp90 and the effect of itraconazole or micafungin in reducing the fungal growth is of great interest as a novel and potential strategy to treat dermatophytoses.

This specific research topic on antifungal drug discovery provides a detailed overview of potential novel antifungal strategies, promising new discoveries and their clinical implications, particularly that of *Candida* species.

## Author contributions

CS and ER contributed to the Editorial.

### Conflict of interest statement

The authors declare that the research was conducted in the absence of any commercial or financial relationships that could be construed as a potential conflict of interest.
